# Enhanced Piezoresistive Cryogel: MWCNT Nanocomposite-Based Wearable Sensors for Real-Time Human Gait and Exercise Monitoring

**DOI:** 10.1021/acsomega.4c10391

**Published:** 2025-01-30

**Authors:** Niranjan Deggenahalli Basavaraju, Vaidehi Basavakumar Roopa, Mathew Peter, Jeevan Medikonda, Saumya Bansal, Pramod Kesavan Namboothiri

**Affiliations:** Department of Biomedical Engineering, Manipal Institute of Technology, Manipal Academy of Higher Education, Manipal, Karnataka 576104, India

## Abstract

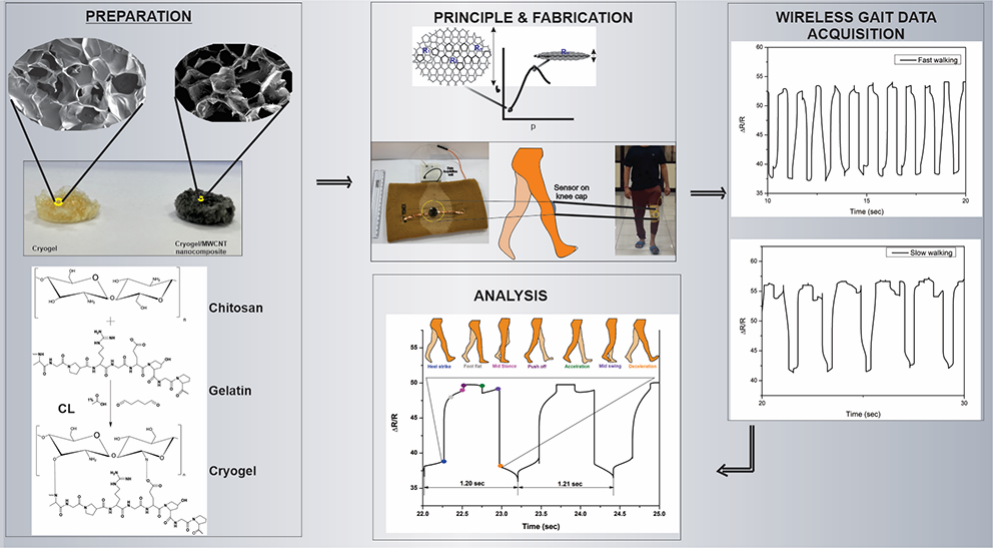

The piezoresistive properties of the modified cryogels were studied. The pressure sensitivity of the cryogel:MWCNT (CG:MWCNT) sensors for different concentrations of MWCNTs are studied. FTIR characterization confirms the formation of CG and CG:MWCNT nanocomposites. Porosity was optimized to enhance the conductivity of the nanocomposite by studying various concentrations. The highly porous structure and the elastic nature of the CG:MWCNT sensors resulted in a change in the electrical percolation even for subtle pressure application, and a linear change in pressure was observed up to 700 kPa. The minimum and maximum pressures detected were 0.4 and 700 kPa, respectively. Electromechanical tests confirm high responsive time and no effect of pore size after stability test, which makes the prepared sensor more span of usage in healthcare conditions. Further, a cryogel:MWCNT wearable wireless sensor module was developed, and the gait signals were acquired wirelessly. The prepared sensor system is able to differentiate between normal, fast, and slow gaits. FFT analysis has been performed to understand the repeatability of signals. Overall, the study emphasizes the potential of the developed sensor system in assisting healthcare professionals, researchers, and individuals in assessing gait characteristics and tracking exercise performance.

## 1 Introduction

In recent years, significant technical advancements have happened in the field of flexible electronics and piezoresistive and capacitive sensors for wearable health monitoring and biomechanical measurement devices. The piezoresistive sensors made from metallic and semiconductor materials have shown lesser displacement strain capacity.^1^^,^^2^ The flexibility, Young’s modulus similar to skin, and thereby maintaining conformal contact with the human body make polymer nanocomposites a suitable material for the development of piezoresistive wearable sensors.^3−5^ Porous 3D gel structures like hydrogels, cryogels, and their composites are considered good candidates to make matrix materials owing to their elastic nature.^6−8^

The porous wearables provide significant improvements in repeatability and sensitivity over thin-film-based sensors because of their unique structural and functional properties.

Porous materials are new types of cryogels that can be synthesized by creating heterogeneous polymer networks through physical and covalent cross-linking. Cryogels typically exhibit interconnected systems of macropores and a sponge-like shape, which facilitate the undisturbed diffusion of solutes across a wide range of sizes. Most of the water content within spongy cryogels is held and can be mechanically extracted by applying pressure. The main features of cryogels are interconnected macroporous structure, elasticity, and mechanical stability.^9−11^ The features of cryogels can be controlled by various factors, such as the temperature, cross-linker percentage, freezing duration, freezing and thawing rate, solvent’s characteristics, and incorporation of soluble and insoluble additives. These features of cryogels make them a suitable matrix for developing strain/pressure sensors.

Multiwall carbon nanotubes (MWCNTs) have been identified as highly notable materials with a wide range of applications, including stretch sensors.^12−14^ The nanotubes comprise multiple concentric layers of graphene sheets rolled into cylindrical shapes, resembling long tubes. MWCNTs possess exceptional physical and chemical characteristics, rendering them highly promising for various applications.^15^^,^^16^ This material’s primary qualities of interest include porosity with a wide surface area and high interfacial adsorption quality. These materials’ outstanding mechanical and electrical properties render them very suitable for developing stretch sensors capable of detecting and quantifying mechanical deformation. Researchers have utilized the distinctive features of MWCNTs to create stretch sensors that exhibit exceptional sensitivity and responsiveness. This is achieved by capitalizing on changes in the electrical conductivity or resistance of MWCNTs when they are subjected to strain. These sensors are utilized in a wide range of fields, including wearable electronics, healthcare devices, robotics, and structural health monitoring.^17−19^ Particularly, in this area, porous nanocomposite sensors, researchers showed a very sensitive detection range as low as 0.022 kPa and so on.^20−22^ However, there are very few studies on optimizing between very low and high detection range. Other authors showed a high detection range at 500 kPa but very few repetitive cycles. In this work, there is optimization in pore size to conductivity to infuse this sensor in multiple applications. On this overview, an article reports that the development of conductive cryogel:multiwall carbon nanotube (CG:MWCNT) composites is for wearable sensors.

### 1.1 Pore Size, Conductivity, and Filler Concentration

During the formation of a MWCNT:cryogel nanocomposite, MWCNTs and polymer interacts by chemical and physical adsorption.^23^ Physical adsorption is the primary interaction between MWCNTs and the polymer matrix of the cryogel. This involves van der Waals forces and π–π stacking interactions between the aromatic rings of MWCNTs and polymer chains. Physical adsorption enables uniform distribution of MWCNTs in the cryogel matrix.^24^^,^^25^ In this work, the concentration of MWCNTs is optimized to tune the conductivity and ensure long-term stability of the cryogel. The cross-linking materials enhance the chemical adsorption between MWCNTs and the polymer matrix. These cross-linking agents chemically interact with hydroxyl (−OH) or amine (−NH_2_) functional groups found on the surface of both MWCNTs and polymer chains.^26^ Chemical bonding occurs when covalent connections are formed between functional groups on the surface of MWCNTs within the polymer matrix. This interaction forms covalent bonds, which firmly attach the MWCNTs to the cryogel matrix.^25^^,^^27^ The presence of chemical bonding improves the stability and mechanical strength of the composite material, hence avoiding detachment or aggregation of MWCNTs during the use. The objective of this study is to develop a cryogel:MWCNT-based wearable sensors for human movement analysis.

## 2 Materials and Methods

The MWCNTs used in this study were produced by the Chemical Vapor Deposition method (CVD), supplied by Adnano Technologies Pvt ltd., Shimoga, India. According to the supplier, the outer diameter of MWCNTs is 10–30 nm, inner diameter 5–10 nm, length >10 nm, and a purity of ∼99%. Chitosanerom shrimp shells (75%), gelatin powder for bacteriology, and glutaraldehyde (25%) for synthesis were procured from Loba Chemie, India. DI Type-III water was used for all experiments.

## 3 Experimental Section

The quantities of chitosan and gelatin are determined based on the calculations. Next, chitosan is added to a beaker containing a measured quantity of 1% acetic acid. The mixture is then placed on a magnetic stirrer and heated at 40 °C until the chitosan completely dissolves. After dissolution, gelatin is added and held on the stirrer until it completely dissolves. A solution of glutaraldehyde with a defined concentration will be prepared by dissolving 25% glutaraldehyde in 1% acetic acid.^28^

The chitosan–gelatin solution is poured into molds, along with a small amount of the prepared glutaraldehyde solution. The mixture is then agitated to thoroughly blend the cross-linker and the components. Subsequently, the molds are placed in a freezer set at a temperature of −20 °C for the duration of t. The molds are removed the following day and put in a refrigerator maintained at a temperature of 4 °C for 2 h. Once removed from the refrigerator, the molds are later defrosted in a water bath for approximately 40 min. The cryogels are subsequently purified by cleaning with 0.1% sodium borohydride to remove any remaining glutaraldehyde. This is followed by washing with deionized water and additional cleansing with 70% and 80% methanol to eliminate excess sodium borohydride. The cryogels that have been prepared are allowed to dry in ambient conditions for 24 h.

### 3.1 Cross-Linker Optimization

Glutaraldehyde was employed as the cross-linking agent for the cryogels due to its ability to create stable covalent bonds with the amine group of the chitosan–gelatin polymer. The concentrations of glutaraldehyde were altered to attain an optimum concentration that effectively facilitates the formation of porous and stable cryogels. By obtaining optimized concentrations of glutaraldehyde, cryogels were prepared using concentrations of 0.2%, 0.6%, and 0.8%.

After experimentation and development of cryogels, utilizing the concentrations revealed the subsequent findings: The cryogels with a concentration of 0.2% exhibited excessive porosity but were highly brittle, whereas the cryogels with a concentration of 0.6% had relatively lower porosity but a slightly more rigid structure. The cryogels with a concentration of 0.8% exhibited an optimal firmness and a significant porosity level. Therefore, we have chosen to use a concentration of 0.8% as the primary cross-linker in our subsequent studies.

### 3.2 Chitosan–Gelatin Ratio Optimization

The C:G ratio was tuned by combining it with an optimized concentration of the cross-linker at 0.8%. Cryogels were prepared with cryogel/gelatin ratios of 2:3 and 2:5. The observed mechanical properties were found to be excessively brittle and weak, leading to the decision to abandon further experimentation.

The chitosan amount was kept constant through additional experimentation, while the gelatin ratio was varied. We developed cryogels with 1:3, 1:4, 1:5, and 1:6 ratios while keeping the glutaraldehyde concentration at 0.8%. Mechanical properties were studied to optimize the C/G ratios for further work. Compression tests were carried out for the same. The results revealed that the 1:4 ratio cryogel exhibits the highest bulk modulus, which indicates its superior mechanical strength due to its resistance to deformation and lower compressibility compared to other cryogels. The samples must possess adequate mechanical properties to align with the porous and resilient properties. The mechanical strength of the 1:4 and 1:5 ratios was nearly identical, with bulk moduli of 10.13 and 9.93 kPa, respectively. For further work, the 1:4 ratio of the C/G cryogel was used.

The selected C/G ratio (1:4) cryogel with optimized electrical property and porous structure was used for developing wearable sensors.

### 3.3 Porosity Calculation

The quantification of pore volume in a sample can be achieved by assessing the overall porosity, a significant characteristic of cryogels, as it affects both their mechanical and electrical attributes. The liquid displacement method was employed to determine the porosity of the samples. The dried cryogels of different concentrations were weighed and recorded as *W*_1_. *N*-Hexane was selected as the displacement liquid due to its nonreactive nature, which prevents any swelling or shrinkage of the samples during testing. A small, sealed vessel was obtained and filled with 5 mL of *n*-hexane. The weight of the vessel with *n*-hexane was recorded as *W*_2_. Subsequently, the cryogel was immersed in the vessel containing *n*-hexane for a duration of 5 min at ambient temperature. Following the incubation period, the weight of the container containing the cryogel and hexane was measured and recorded as *W*_3_. Finally, the cryogel was removed, and the weight of the container containing the remaining hexane was measured and recorded as *W*_4_.

The porosity percentage of each sample was determined using the formula
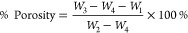


## 4 Results and Discussion

**Figure 1:**
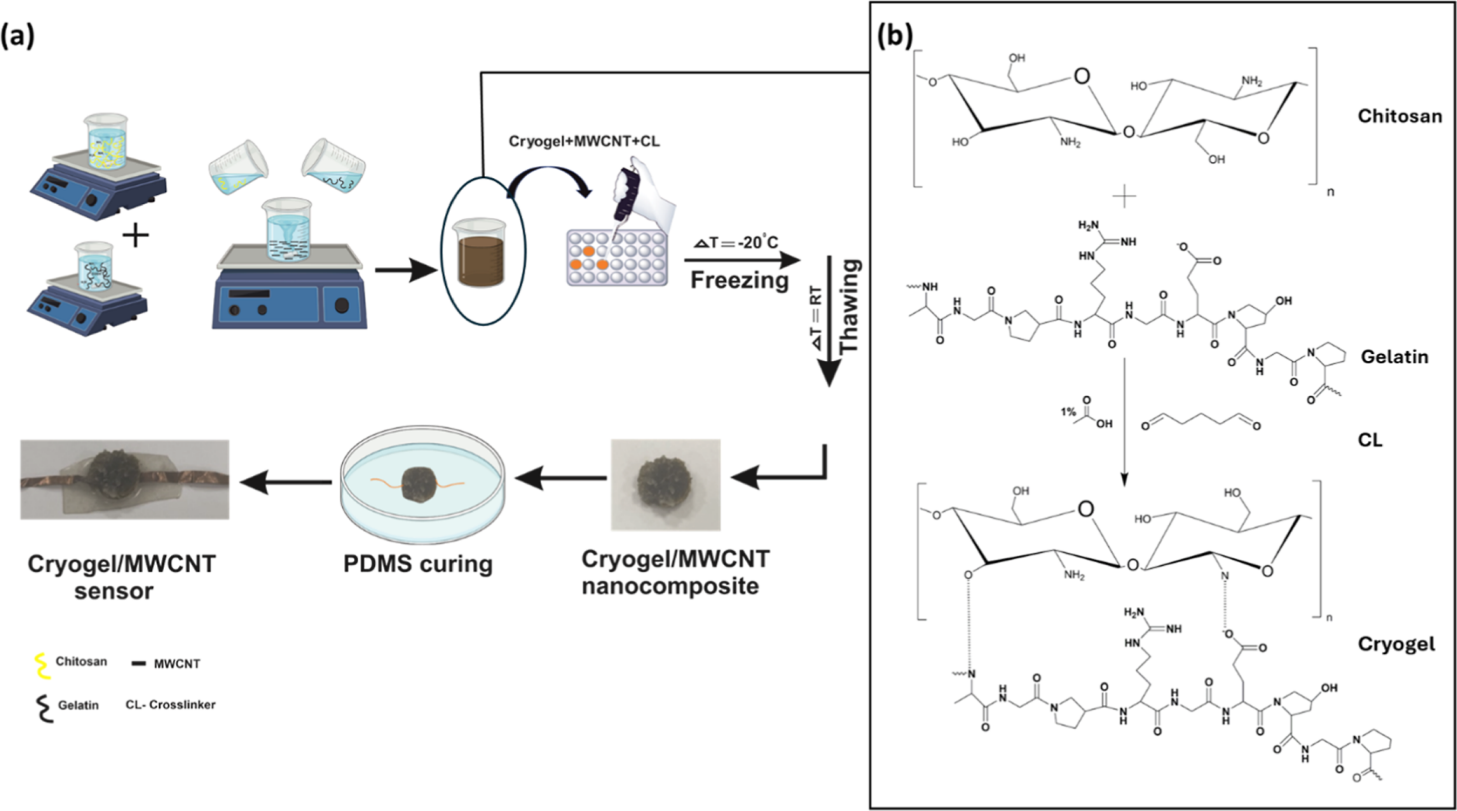
(a) Methodology to prepare Cryogel: MWCNT nanocomposite. (b) Schematic representation of cross-linking between chitosan–gelatin polymer precursors using glutaraldehyde as a nonzero length cross-linker.

A CG:MWCNT-based wearable sensor was prepared by cryogelating gelatin and chitosan with glutaraldehyde as the cross-linker and MWCNT as the nanofiller (Figure 1). First, gelatin and chitosan were stabilized in distilled water and mixed at room temperature. MWCNT was dispersed uniformly in a gelatin-chitosan mixture, and the cross-linker was added for gelation. After the cross-linker was added, the mixture was poured into molds. The cross-linker-added gelatin–chitosan–WCNT mixture was frozen under −20 °C for 24 h and thawed at room temperature. The formed porous nanocomposite was placed on PDMS resin, which was cured to form the elastomeric substrate. As shown in Figure 1a, the copper tapes were inserted to make electrical contacts. Figure 1b shows the schematic of cross-linking between chitosan–gelatin polymer precursors using glutaraldehyde as a nonzero length cross-linker.

### 4.1 FTIR Characterization

**Figure 2:**
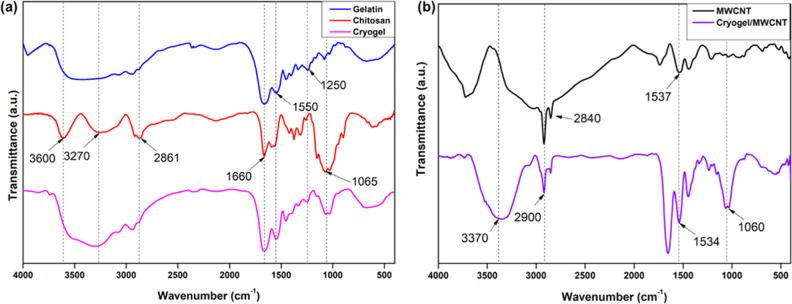
FTIR characterization of (a) gelatin, chitosan, and cryogel (b) shows the MWCNT and CG:MWCNT.

Figure 2a shows that gelatin and chitosan have hydroxyl groups that usually show up as wide bands at 3600 cm^–1^.^29^ Peaks in this region indicate stretching vibrations of hydroxyl groups contributing to hydrogen bonding interactions in the cryogel matrix. Chitosan’s amine groups (−NH_2_) peak at 3270 cm^–1^ in the spectrum, indicating N–H stretching vibrations. This peak suggests that chitosan is present in the cryogel matrix. Gelatin exhibits peaks associated with amide bands (I, II, and III) that correspond to peptide bonds, comprising C=O stretching vibrations (amide I) at 1660 cm^–1^, N–H bending, and C–N stretching vibrations (amide II) at 1150 cm^–1^, and combined C–N stretching and N–H bending modes (amide III) at 1250 cm^–1^. These peaks indicate the presence of gelatin in the cryogel matrix and offer insight into the secondary structure. Gelatin and chitosan contain carbonyl groups (C=O), which usually appear as peaks at 1650 cm^–1^ in the spectrum. The peaks represent the stretching vibrations of C=O bonds in the amide groups of gelatin and the acetamide groups of chitosan. Amide band shifts and variations in hydroxyl group peak intensities may suggest hydrogen bonding interactions between gelatin and chitosan molecules in the cryogel matrix, which indicates the formation of the chitosan–gelatin cryogel matrix.^30^Figure 2b shows the cryogel and Cryogel:MWCNT nanocomposite FTIR results. The IR spectrum of MWCNTs shows the absorption peak at 2900 cm^–1^, attributed to the CH_2_ stretch. The band at 1534 cm^–1^ is attributed to the C=O stretch, and 1060 cm^–1^ is assigned to the C–O stretch. Above all, the bands mentioned above are also present in CG:MWCNT, which confirms the formation of nanocomposite.

### 4.2 Porosity and Electrical Characterization

**Figure 3:**
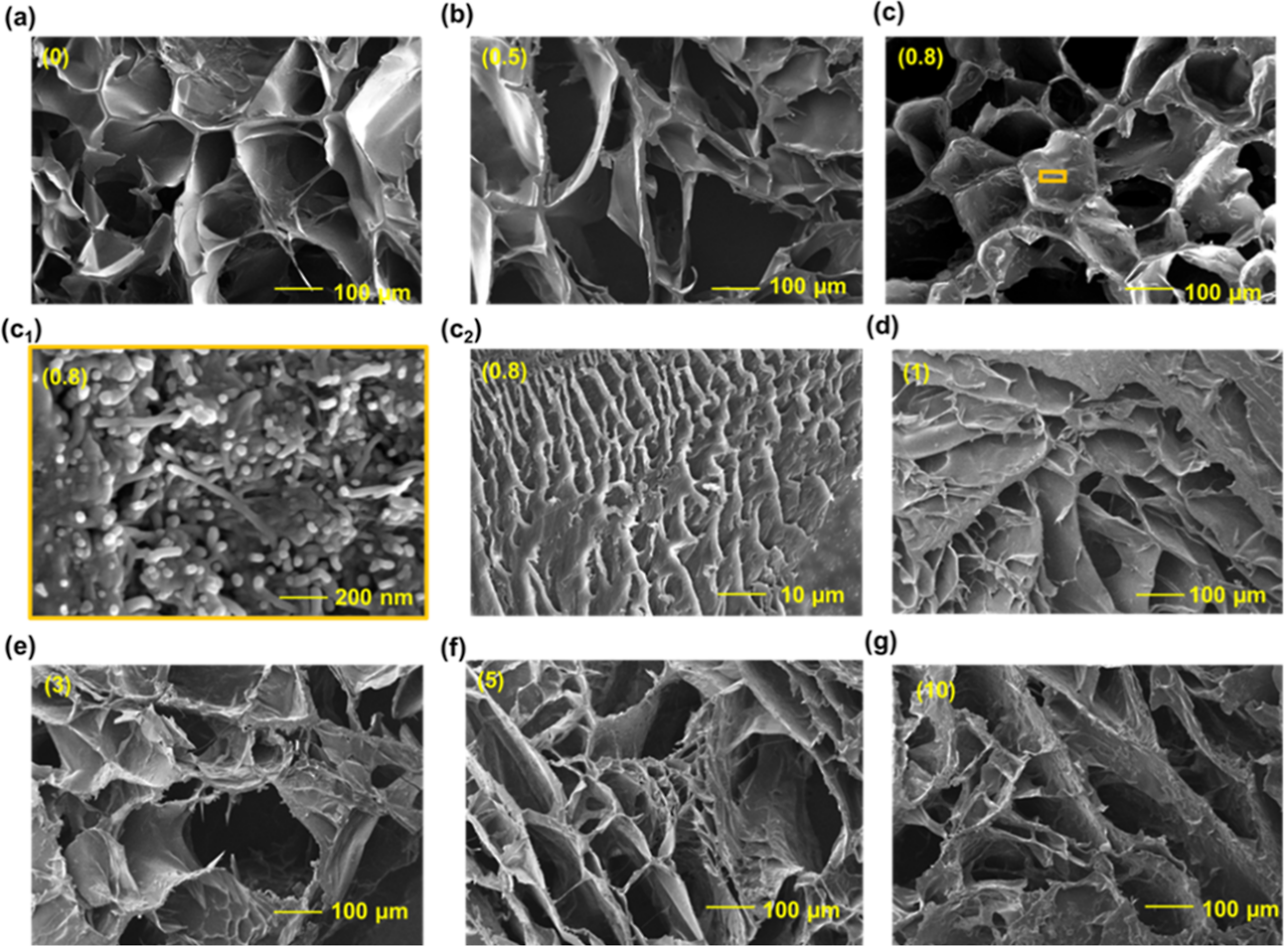
SEM images of the cryogel (a) and CG/MWCNT nanocomposites (b–g) of different MWCNT concentrations.

Figure 3a–g depicts the SEM images of CG:MWCNT nanocomposites with different concentrations of MWCNTs (0%, 0.5%, 0.8%, 1%, 3%, 5%, and 10%). Figure 3c_1_ shows an enlarged view of the CG:MWCNT nanocomposite with 0.8% of MWCNTs dispersed. Figure 3c_2_ shows the CG:MWCNTs with 0.8% MWCNT nanocomposite under compression. Due to compression, the interconnecting networks of cryogel collapse and close the pores; upon releasing the compressed structure, it reforms to its original shape. In Figure 3, SEM images show a reduction in the diameter of the pores with an increase in MWCNT wt % concentration. This makes the nanocomposite less elastomeric, as it reduces the pore volume. The conductivity of CG:MWCNT nanocomposites could be from three components: 1. Cryogel matrix in this study, the cryogel matrix is insulating, and will not contribute to conductivity, 2. water content may contribute as ionic conductivity by filling in the pores of the matrix. In this study, water used was DI water and pores were formed in the insulating matrix, the contribution of water content to the overall conductivity of the CG:MWCNT would be minimal. 3. Carbon nanotubes, which act as conducting fillers in the cryogel matrix. The percolating network of MWCNTs through the polymer interconnecting network in the cryogel matrix significantly contributes to the conductivity of the CG: MWCNT nanocomposite.

**Figure 4:**
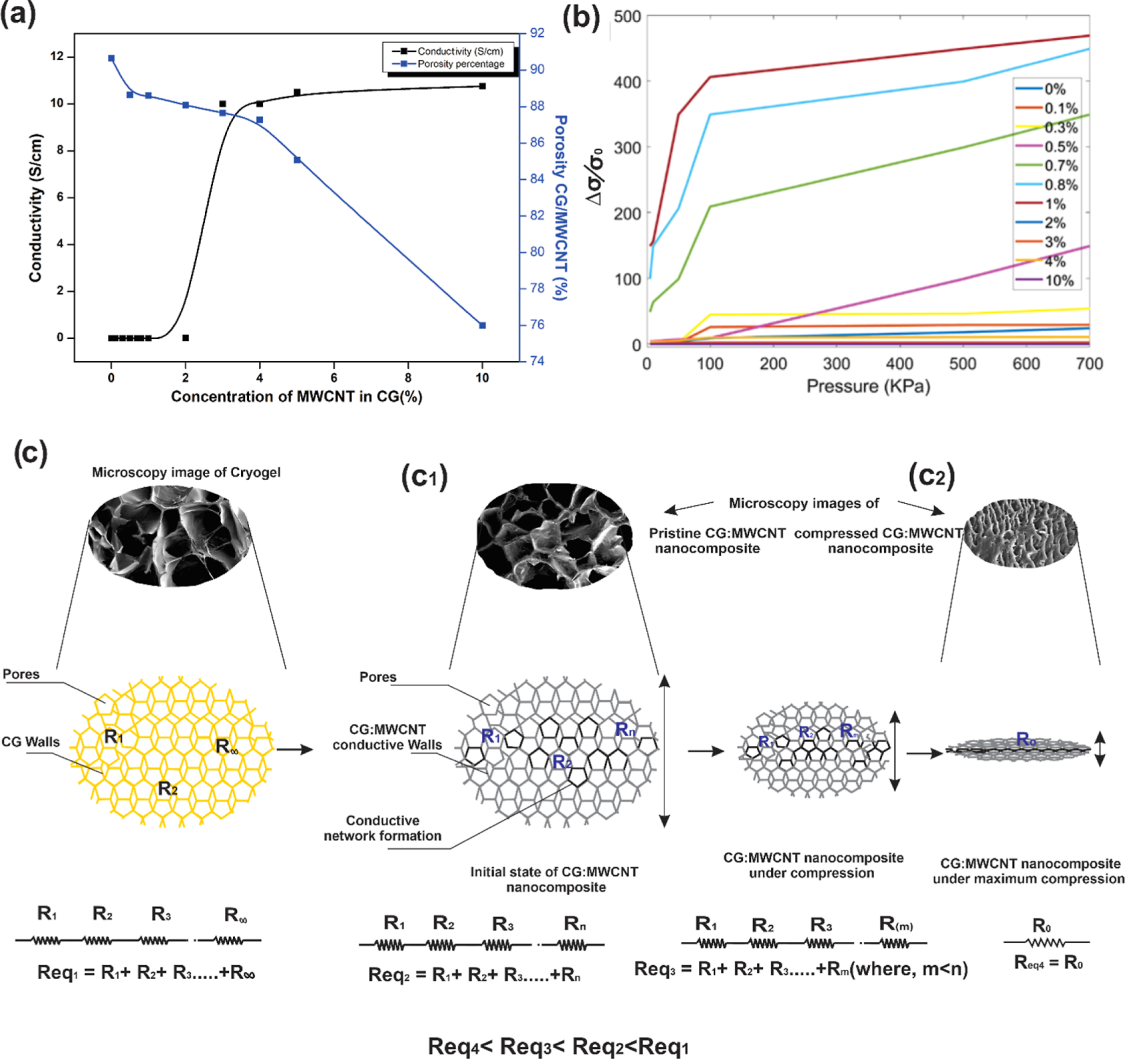
(a) Shows porosity variation compared to the percolation curve, (b) shows the change in the conductivity of the CG:MWCNT nanocomposite of varied MWCNT concentration with applied pressure, and (c–c_2_) illustrates the compression loading for nanocomposites attributing on resistance change in the nanocomposite.

Figure 4a explains the relationship between porosity, conductivity, and electrical percolation of the nanocomposite. As the concentration of MWCNTs in cryogel increased, the porosity of CG:MWCNTs was observed to be decreasing. The porosity of the prepared CG:MWCNT nanocomposites was calculated using the liquid displacement method. Around 14% of reduction in porosity was observed as the MWCNT concentration increased to 10 weight % concentration. This phenomenon could be attributed to interaction between MWCNTs and the cryogel matrix during the freeze and thaw cycle. An increase in the rate of change of porosity above the electrical percolation threshold of MWCNTs in the cryogel matrices was observed. Above the electrical percolation concentration threshold, MWCNTs would be forming continuous networks in the cryogel and the electrical conductivity increases to a maximum and saturates. This continuous network of MWCNTs in a cryogel matrix above percolation threshold would provide mechanical stability to the polymer network, thereby restricting ice crystal growth during freezing, resulting in less porosity in the cryogel matrix. Figure 4b portrays the change in the conductivity of CG:MWCNT with different MWCNT concentrations and pressure. The electrical percolation threshold point was obtained at 1%. The electrical conductivity was observed to saturate after 4 wt % of MWCNT concentration. When pressure is applied, the CG:MWCNT cryogel deforms, and the distance between the MWCNTs in the cryogel matrix reduces, resulting in increased percolation and electrical conductivity. At lower concentrations of MWCNTs in the cryogel matrix, even after the application of pressure to the cryogel and its deformation, the change in distance between MWCNTs was not enough to develop a percolated network and an increase in conductivity. This could be observed from Figure 4b for CG:MWCNT nanocomposites with the MWCNT concentration below 0.5 wt %. In the case of CG:MWCNT nanocomposites with the concentration of MWCNT above 4 wt %, the MWCNTs are highly percolated and the electrical conductivity was saturated. Therefore, application of pressure to the CG:MWCNT nanocomposites above 4 wt % of MWCNT concentration also could not result in a considerable change in the electrical conductivity. As the applied pressure increased to 90 kPa, there was a sharp increase in the electrical conductivity of the CG:MWCNT nanocomposites. This could be attributed to the major collapse of the porous structure in the cryogel matrix as Figure 4c. Above 90–100 kPa, a linear response to an increase in the applied pressure was observed. Figure 4c illustration corresponds to the relation of electrical percolation of the CG:MWCNT nanocomposite. Initially without addition of filler MWCNT, there will be no conduction in the nanocomposites as shown in Figure 4c. It can be considered as infinite number of resistors, from *R*1 to R∞, connected serially resulting in very high resistance. After the introduction of MWCNTs at a near percolation threshold, the total resistance reduced due to the formation of the conductive path. This could be denoted as a serial arrangement of finite number of resistors, *R*_0_ to *R*_*n*_, which results in a finite measurable resistance as shown in Figure 4c_1_. In this condition, electron transport may happen due to ohmic contacts and tunneling. Once load is applied, the tunneling path collapses, and more of ohmic contact based conductivity will appear in the cryogel nanocomposite. This can be attributed to the collapsing of pores which eliminates resistance paths and brings more conductivity due to application of load. Then, it could be represented as the total number of resistors arranged in series reduces from *n* to *m*, where *n* > *m*, as shown in Figure 4c_2_. At maximum compression, all resistance paths broken create a high conductivity path. This denoted as single resistance “*R*_o_”, as shown in Figure 4c_2_. If considering the equivalent resistance at each stage, equivalent resistance *R*eq_4_, resistance at maximum compression is less than Req_3_, which is resistance at moderate compression. *R*eq_3_ is lesser than *R*eq_2_, which is resistance at pristine condition of the CG:MWCNT nanocomposite. *R*eq_2_ is lesser than *R*eq_1_, which is a resistance CG matrix.

### 4.3 Fabrication of Sensors

Considering the better pore size, conductivity (1 S cm^–1^), and porosity, CG:MWCNT with 0.5 wt % of MWCNT concentration was selected for further experiments.

This prepared CG:MWCNT wearable sensor is attached to an in-house prepared data acquisition system (DAU). The circuit diagrams for acquiring signals are shown in the Supporting Information (Figure S1). The DAU involves the AVR microcontroller (Arduino nano), battery to power the microcontroller, and Bluetooth module for wireless data transmission. DAU was designed using an Arduino nano (ATPmega328P) processor with a voltage divider program. MegunoLink was used to acquire wireless signals from the nanosensor. The sampling frequency of 128 points was used for acquiring signals. A wearable sensor was attached to knee brace by a double-sided tape. Knee brace wear to left knee. While walking, there will be bending and swinging of kneecaps. This would result in increases in conductivity. Similarly, while coming to the original shape, conductivity decreases. This mechanism of change in the electrical conductivity of nanocomposites is used to acquire the movement of body part.

### 4.4 Electromechanical Test Characterizations

**Figure 5:**
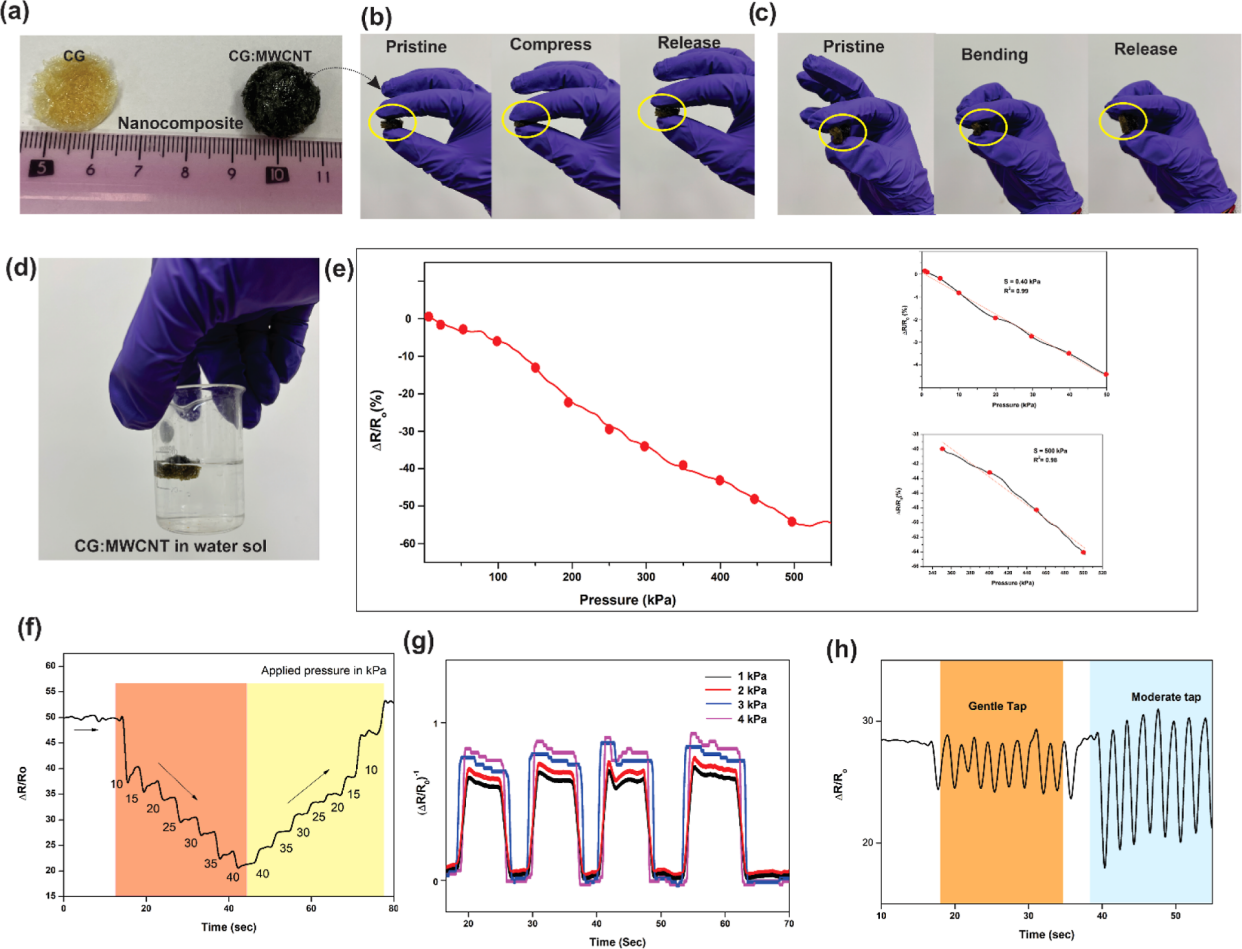
(a) Shows the pictorials of CG and CG:MWCNT nanocomposites, (b,c) shows macrotransformations compression and bending, respectively, (d) displays the nanocomposite in a water solvent, (e) displays the graph of compression loading for sensitivity test, (f) demonstrations of graph of incremental loading on sensor, (g) shows the graph of repetitive loading of multiple cycles on the sensor, and (h) picture shows the demonstration of the sensor on gentle and moderate tap.

The prepared sensor was investigated for electromechanical tests to understand the performance of the sensor. Figure 5a shows the nanocomposites of CG and CG:MWCNT. Figure 5b,c shows that the prepared CG:MWCNT nanocomposite sensor performs pristine to compressed condition and pristine to bending condition, respectively. This sensor shows that MWCNTs are not pulling out of water, which makes them washable, which is shown in Figure 5d. To understand sensitivity, compressive pressure has been applied until 500 kPa. During that, majorly two liner curves appeared, which suggests that sensitivity at 0.4 and 500 kPa detected at a compressive pressure. Figure 5e describes the compressive loading at incremental cycles. Figure 5f displays compressive loading at incremental and releasing cycles, which confirms that the sensor is able to detect incremental loading and coming back to preceding position after releasing of load. Another test of a minimal load of 1 to 4 kPa applied consecutively for multiple cycles. The sensor is able to detect and differentiate on small loads, as shown in Figure 5g. To understand very low detection range, tap test was performed on a sensor; the results show that the sensor is able to detect gentle tap (≈1 Hz) to moderate tap (≈3 Hz) which is shown in Figure 5h.

**Figure 6:**
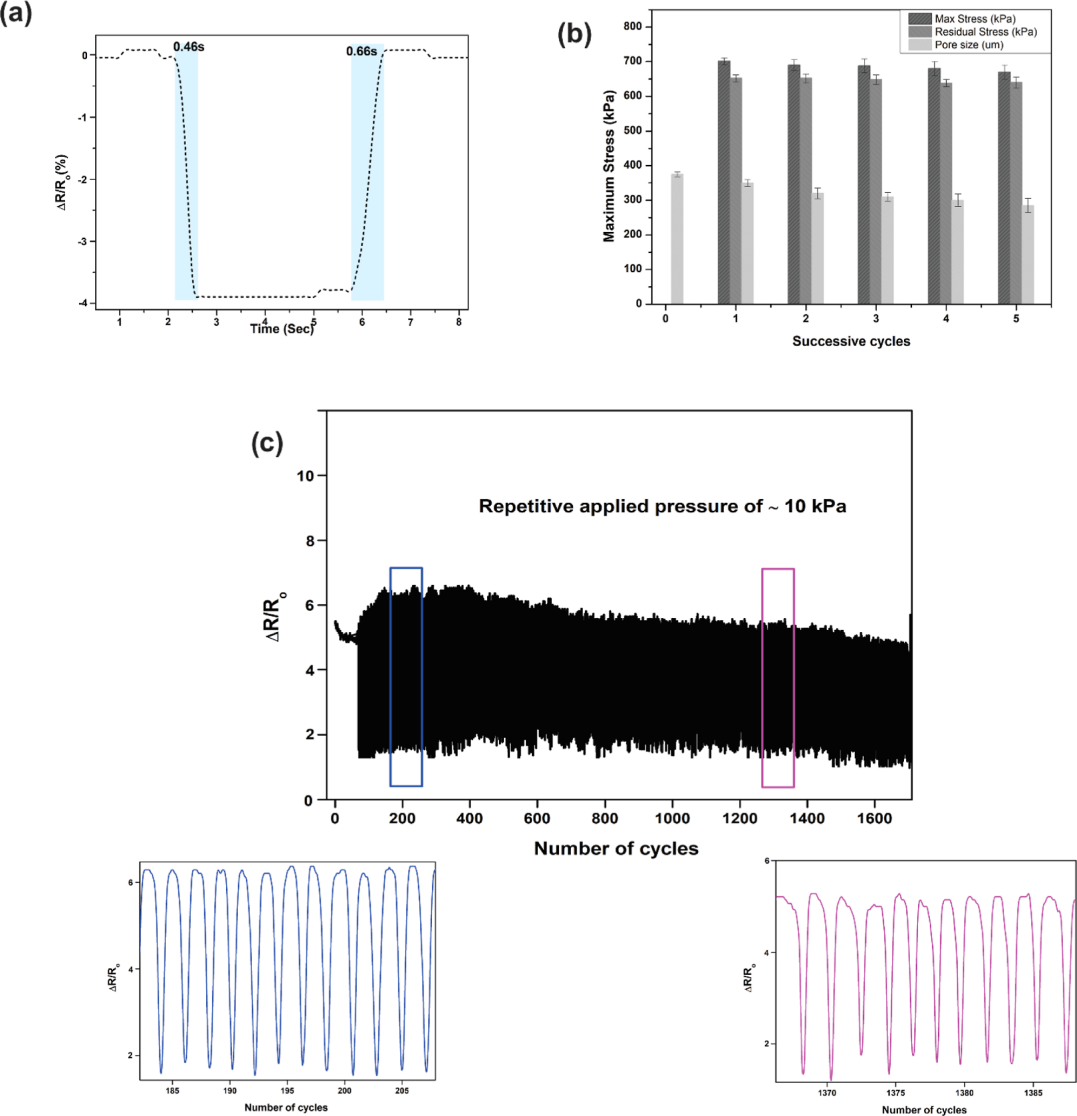
(a) Shows the responsive time of the sensor on loading and release points, (b) shows the comparison graph of maximum stress on successive cycles and its attribution on pore size of sensor after test (c) depicts the stability of sensor and its enlarged view at different cycles.

This prepared CG:MWCNT sensor-responsive time was highly sensitive; while applying load, the sensor performed at 0.46 and 0.66 s for loading and releasing, which is shown in Figure 6a. To understand the repetitiveness of the sensor, a maximum load of 700 kPa was applied for multiple cycles as shown in Figure 6b. Sensors are able to detect the same load on successive cycles. Correspondingly, poresize characterization was performed to analyze any deformational changes due maximum load. Figure 6b confirms that, after loading and releasing, the influence on pore size was very minimal. To understand the stability of the sensor, consecutive loading of ≈10 kPa applied for more than 1000 cycles, which is shown in Figure 6c. The inset figure explains the initial loading and final loading differences very minimal. Micrographs attached in supplementary work for before and after stability test. These above results show that the prepared CG:MWCNT sensor can be used for highly sensitive applications such as tapping to large movement applications such as Gait acquisition.

### 4.5 Applications

**Figure 7:**
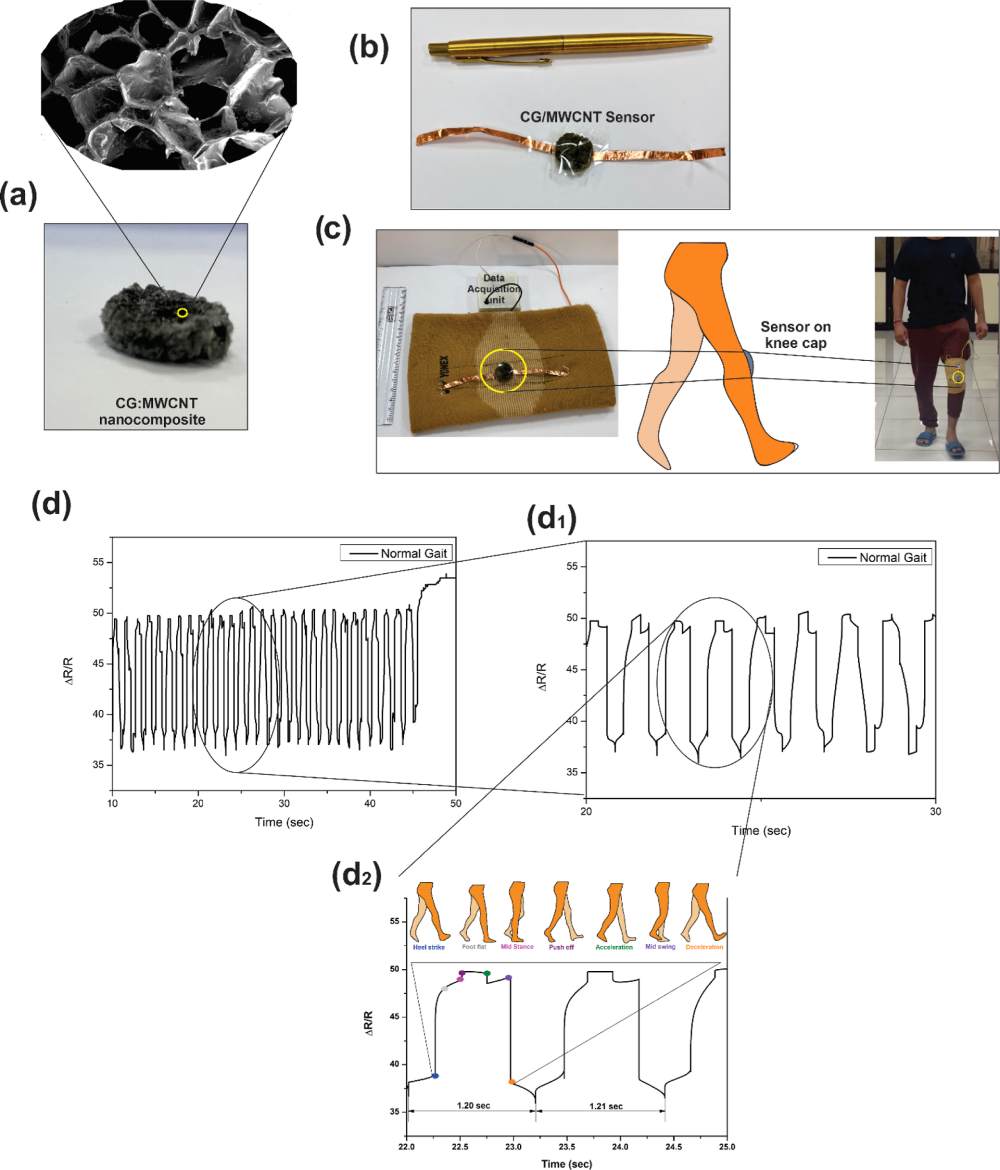
(a) CG:MWCNT nanocomposite (b) with DAU and sensor (c) attached on knee cap, (d) acquired normal or regular signals from the sensor, (d_1_) shows the enlarged view of the same which depicts, and (d_2_) gait characteristics.

Figure 7 d shows gait data acquired by the CG:MWCNTs wearable sensor. The *x*-axis of the figure represents time in seconds, and the *y*-axis represents ratio changes in resistance (Δ*R*/*R*_o_). All signals were acquired for 1 min, and to analyze, 10 s of the signal was chosen. Data was acquired for one min on a straight path with different paces of regular/normal walk, fast walking, slow walking, and very slow walking. Along with gait data, jumping and squat exercises were also recorded to test the sensors in different environments.

**Figure 8:**
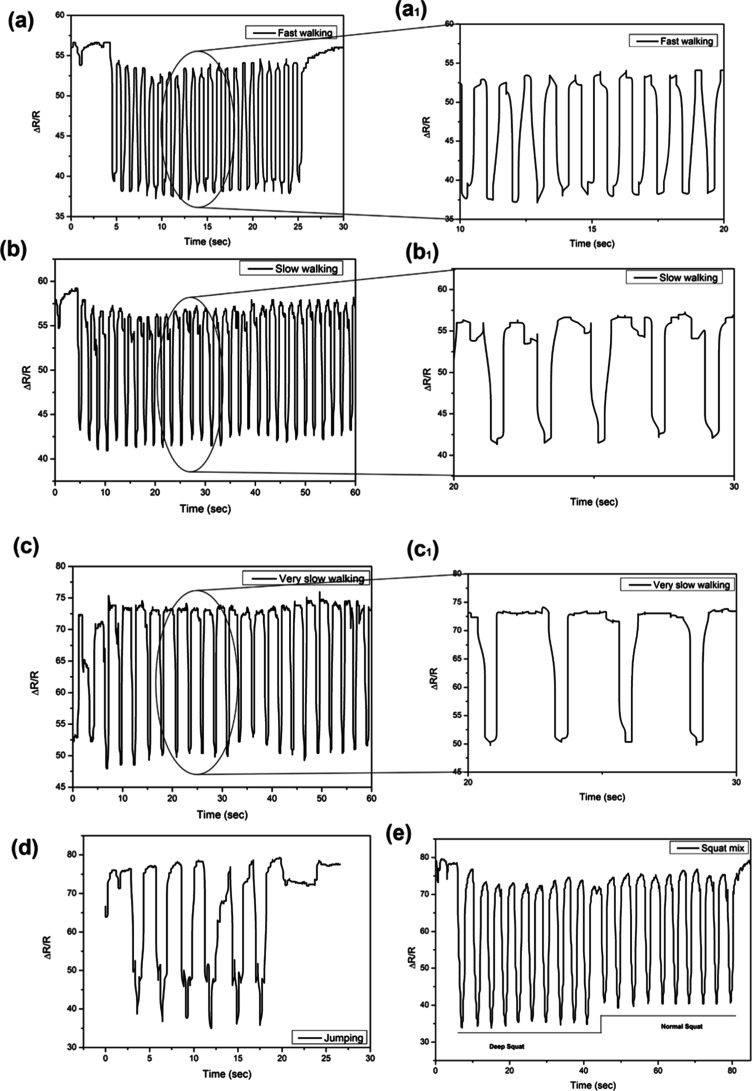
Acquired gait signals from the CG:MWCNT sensor of (a) fast gait and (a_1_) its enlarged view, (b) slow gait and (b_1_) its enlarged view, (c) very slow gait and (c_1_) its enlarged view, and exercises such as (d) jumping and (e) squats.

Figure 7d shows the normal gait data acquired by the sensor. Figure 7d_1_ depicts an enlarged view of a normal gait of 10 s. As shown in Figure 7d_2_, the time required to complete one gait cycle was 1.2 s, that is, the first heel strike to push off of the same leg, which is shown in the enlarged image of normal gait. This prepared sensor could detect gait phases, stance phase (heel strike > foot flat > mid stance), and swing phase (push off > acceleration > mid swing > deceleration), which are shown in the enlarged normal gait image Figure 7d. Figure 8a,a_1_ shows the signals acquired during fast gait and its enlarged view, respectively. From this figure, the time required to complete one cycle for fast gait was 0.75 s. In a fast gait, the width of one cycle is reduced compared to a normal gait. While performing a fast gait, heel strike to foot flat time will be much less; this could be observed from the enlarged image of fast gait, shown in Figure 8a_1_. Similarly, the slow gait cycle acquired from the sensor is shown in Figure 8b and its enlarged view in b_1_, which shows that the time to complete one gait cycle increased to 2 s. The time required to finish each gait phase increased in the slow gait cycle compared with normal and fast gait cycles. Similarly, a very slow gait cycle, as shown in Figure 8c and its enlarged view in c_1_, data acquired from the sensor. In this action, very slow walking for 60 s data was acquired. The pitch for completing one gait cycle is enlarged, and it takes 2.5 s to complete one cycle of very slow gait. These results show that gaits from normal, fast, slow, and very slow gaits can be recorded using the developed sensor. The prepared system is able to differentiate among all different gaits. Apart from the gait cycle acquisition, the sensor could acquire strength exercises such as jumping and squats, as shown in Figure 8d,e, respectively. The sensor was attached to the knee for this experiment, and the data was acquired. Figure 8d shows jump squat exercise data. These data show repetitive signals for jumping. Figure 8e shows the squat exercise for normal and deep squats. For the deep squat, the *y* range varies 42 points; for normal, it varies to 33 points. This shows that the prepared sensor can differentiate between deep and normal squats. A working prototype’s demonstration of signal acquisition is shown in supplementary videos.

**Figure 9:**
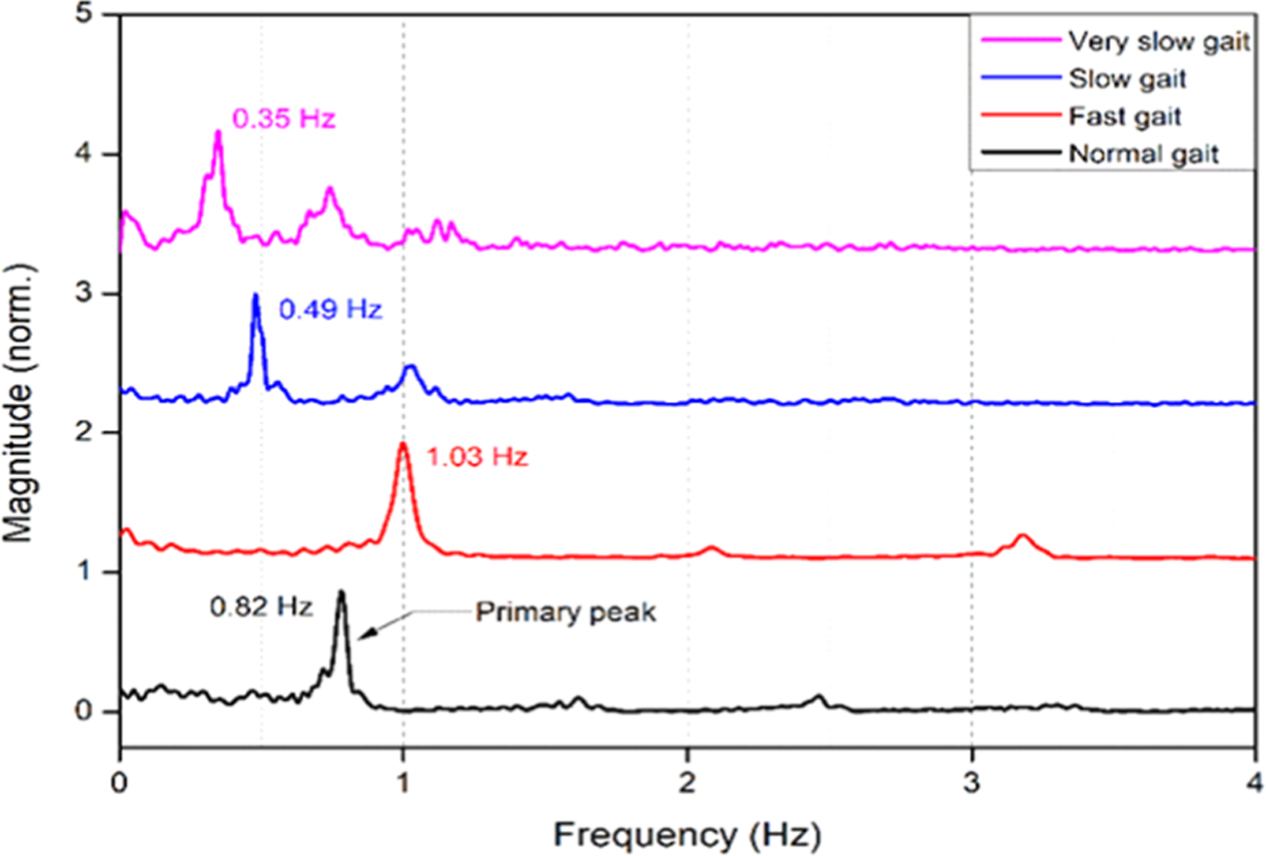
Frequency change for different gaits.

The acquired time domain signals were converted to frequency domain by carrying out fast Fourier transform (FFT) of the signals to understand periodic behaviors of the gait patterns.^31,32^Figure 9 shows the frequency versus magnitude graph of different gaits, including regular, fast, slow, and very slow gaits. In this figure, for a regular gait, the primary peak appears at 0.82 Hz. Other additional peaks represent harmonics due to overtones of the gait frequency. The frequency observed in the fast gait was 1.03 Hz. For the slow and very slow gaits, the frequency was observed at 0.49 and 0.35 Hz, respectively. The progression from slow to fast gait shows that data accurately captures a range of gait speeds. The variation in frequency across the different walking speeds indicates regular human gait dynamics, with slower speeds producing lower frequencies. Using the above data (time-domain and frequency-magnitude signals), we studied walking dynamics parameters. This includes Stride rate (Cadence)^33^^,^^34^
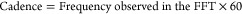


The peak frequency observed for a normal gait in FFT was 0.84 Hz. Using the above formula, the cadence for a normal gait observed was 50.4 steps per minute. Similarly, for a fast gait, 61 steps were observed/min for slow and very slow gaits, 29 and 21 steps/min. For more analysis, by considering a regular gait, the gait characteristics of a healthy subject covered a distance of 9 m in 9.5 s, yielding gait speed at 0.947 m/s. From clinical norms, this data usually falls between 1 and 1.5 m/s. This gait speed may signify a regulated walking pace or represent the specific circumstances of the testing environment. The stride length was calculated at approximately 1.127 m. The following equation is used to calculate the stride length.^35^
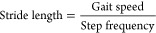


The average stride length for a healthy adult is around 1.1 to 1.5 m. A stride length of 1.127 m is within the range of normal values. This is a sign of efficient walking, as the stride length is near the average. The FFT analysis of the gait data identified a significant peak at 0.84 Hz, signifying a rhythmic and consistent gait cycle. Minor peaks between 2.5 and 3.5 Hz indicate supplementary harmonic content linked to footfalls, further demonstrating a stable gait pattern.

## 5 Conclusions

The study showcased the development of CG:MWCNT nanocomposite-based sensors and its ability to capture and analyze a wide range of gait patterns and strength exercises. Figures 7 and 8 comprehensively analyze various gaits, highlighting their unique characteristics, such as cycle times and phase durations. This highlights the sensor’s ability to identify between various gait speeds and accurately recognize specific phases. In addition, the sensor demonstrated its versatility by expanding its capabilities beyond gait analysis to involve strength exercises like jumping and squatting. Figure 8d,e demonstrates the sensor’s capacity to record repetitive signals during jumping exercises and differentiate between normal and deep squats by identifying subtle variations in data patterns.

Overall, the study emphasizes the potential of the developed sensor system in assisting healthcare professionals, researchers, and individuals in assessing gait characteristics and tracking exercise performance. These findings enhance the comprehension of gait dynamics in regulated environments and highlight possible factors influencing walking patterns. Further developments and uses of this technology could greatly improve rehabilitation protocols, enhance sports training programs, and facilitate remote health monitoring systems.

## Supporting Information Available

Walking demo, sensitivity of a sensor, and sensor demonstration at 50 °C (AVI)
Circuit diagram, micrographs before and after test, program used for acquiring signals uploaded in the document (PDF)

## Supplementary Material

ao4c10391_si_001.avi
