# Supplementary material for: Enhanced Piezoresistive Cryogel: MWCNT Nanocomposite-Based Wearable Sensors for Real-Time Human Gait and Exercise Monitoring
Source: ACS Omega. 2025 Jan 30;10(5):4940–51. doi: 10.1021/acsomega.4c10391 (PMC11822496; doi:10.1021/acsomega.4c10391)
Supplement: Supplementary file 2 — ao4c10391_si_002.pdf [file ao4c10391_si_002.pdf]

Enhanced Piezoresistive Cryogel: MWCNT nanocomposite-based wearable sensor for real-time human gait and exercise monitoring

Niranjan Deggenahalli Basavaraju[a], Vaidehi Basavakumar Roopa[a], Mathew Peter[a], Jeevan Medikonda[a], Saumya Bansal[a] and Pramod Kesavan Namboothiri[a*]

[a]Department of Biomedical Engineering, Manipal Institute of Technology, Manipal Academy of Higher Education, Manipal 576104

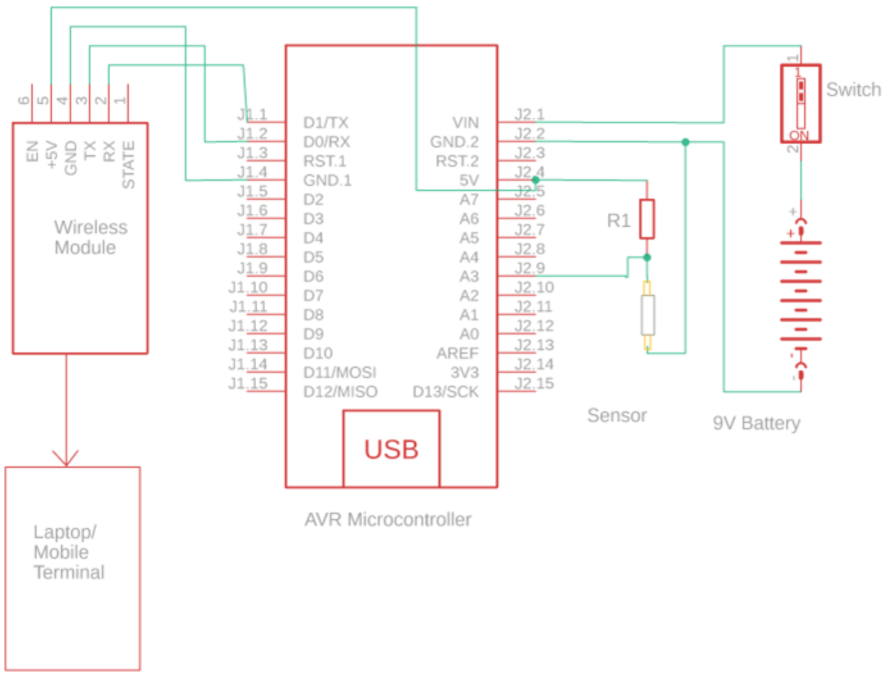

Figure S1: Circuit diagram for Wireless data acquisition

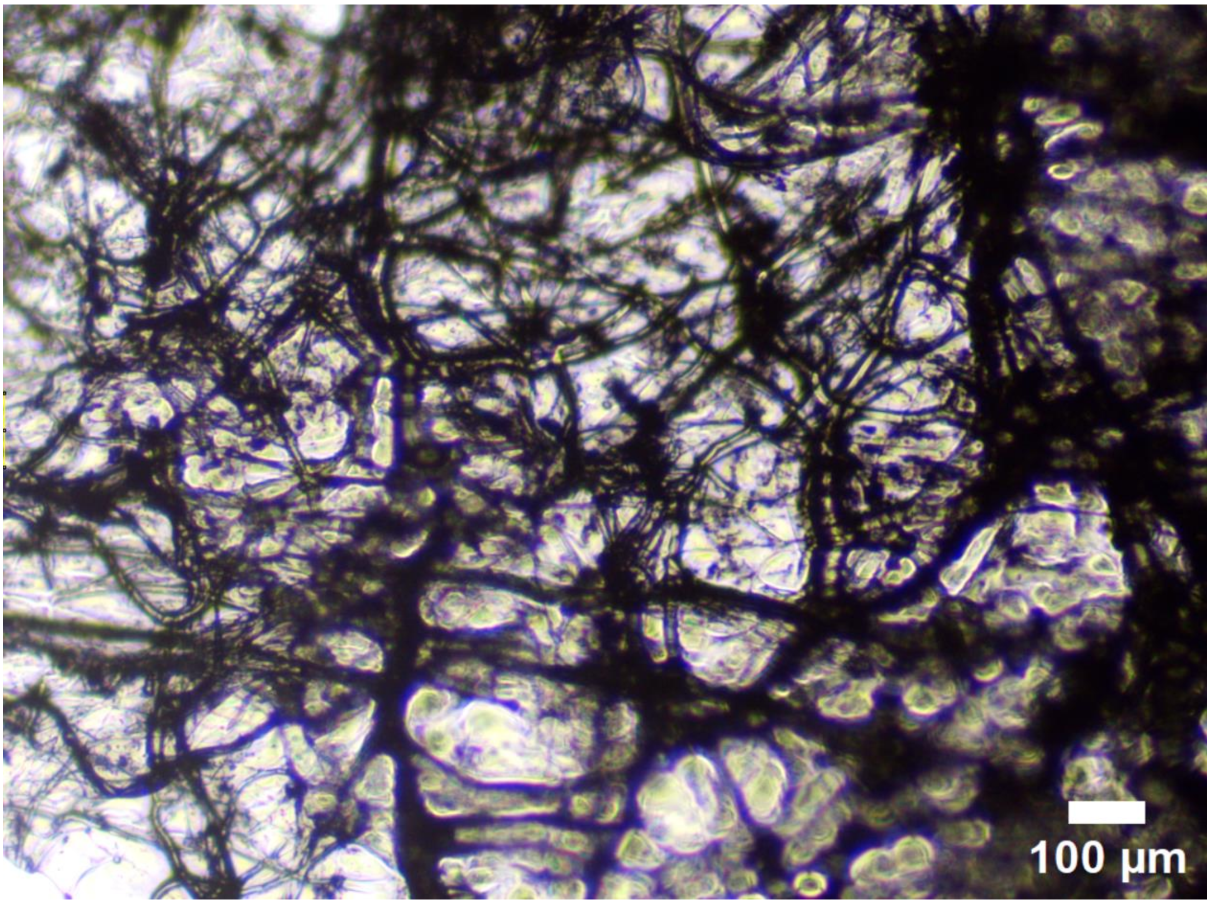

Figure S2: Before stability test

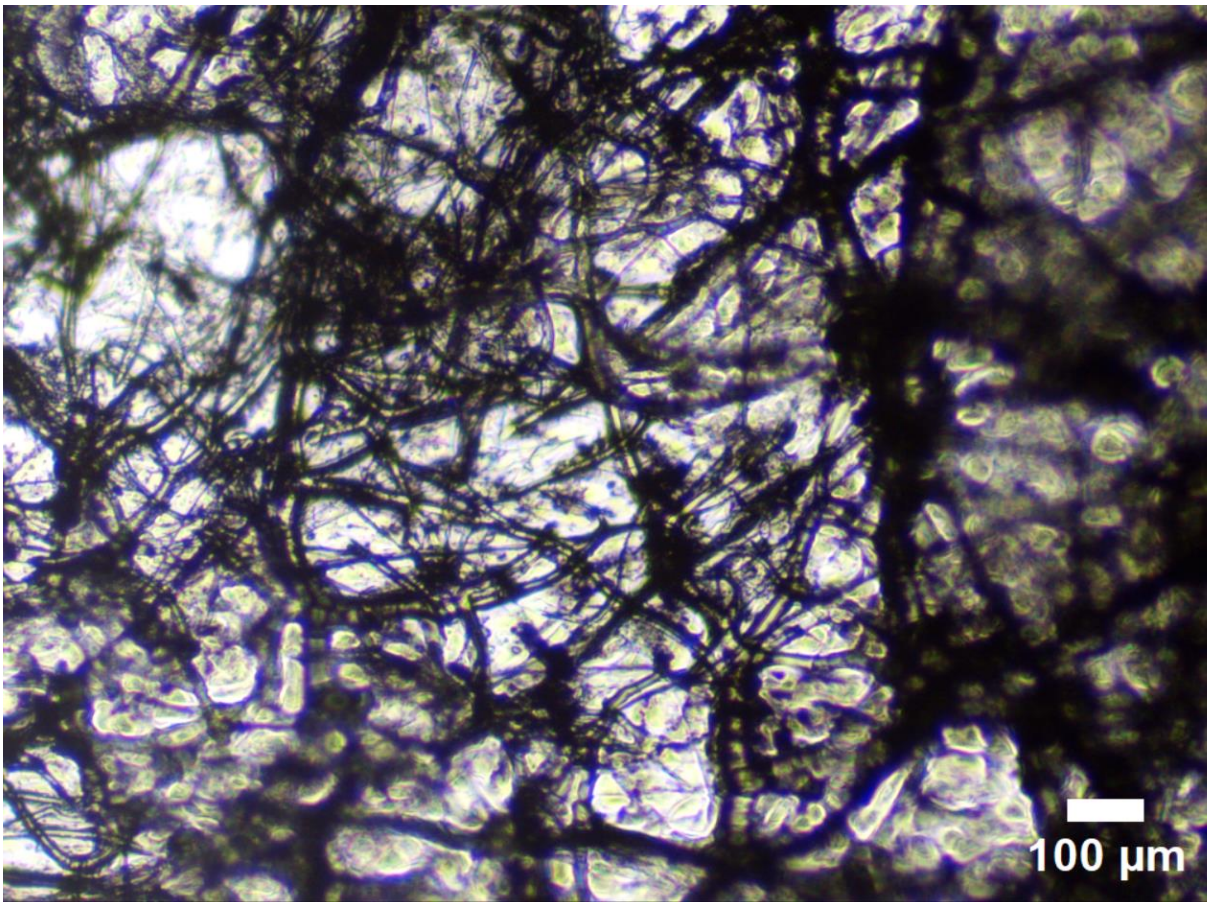

Figure S3: After stability test

Voltage divider program for acquiring resistance change in Cryogel/MWCNT sensor

```
int analogPin = A1;
int raw = 0;
int Vin = 5;
float Vout = 0;
float R1 = 100;
float R2 = 0;
float buffer = 0;

#include "MegunoLink.h"
TimePlot MyPlot;
void setup()
{
Serial.begin(9600);
}

void loop(){
//  for (int i=0; i<1000; i++){
  raw = analogRead(analogPin);
  //}
  if(raw)
  {
    buffer = raw * Vin;
    Vout = (buffer)/1024.0;
    buffer = (Vin/Vout) - 1;
    R2= (R1 * buffer)/(100);
   // Serial.print("Vout: ");
    //Serial.println(Vout);
    Serial.print("R2: ");
    Serial.println(R2);
   MyPlot.SendData("X", R2);
   delay(0);
  }
}
```
